# Community composition of black flies during and after the 2020 vesicular stomatitis virus outbreak in Southern New Mexico, USA

**DOI:** 10.1186/s13071-024-06127-6

**Published:** 2024-02-27

**Authors:** Madelin J. Whelpley, Lawrence H. Zhou, Jeremy Rascon, Bailey Payne, Brett Moehn, Katherine I. Young, Chad E. Mire, Debra P. C. Peters, Luis L. Rodriguez, Kathryn A. Hanley

**Affiliations:** 1 Department of Biology, College of Arts and Sciences, New Mexico State University, Las Cruces, NM USA; 2 Department of Biological Sciences, University of Texas El Paso, El Paso Texas, USA; 3 United States Department of Agriculture, Agricultural Research Services, National Bio and Agro-Defense Facility, Foreign Arthropod-Borne Animal Diseases Research Unit, Manhattan, KS USA; 4 United States Department of Agriculture, Office of National Programs, Beltsville, MD USA; 5 United States, Department of Agriculture, Agricultural Research Services, Plum Island Animal Disease Center and National Bio- and Agro-Defense Facility, Manhattan, KS USA

**Keywords:** Simuliidae, Livestock, Vesicular stomatitis virus, *Simulium robynae*, *Simulium meridionale*, *Simulium mediovittatum*, New Mexico, Rio Grande

## Abstract

**Background:**

Vesicular stomatitis virus (VSV), a vector-borne pathogen of livestock, emerges periodically in the western US. In New Mexico (NM), US, most cases occur close to the Rio Grande River, implicating black flies (*Simulium* spp.) as a possible vector. In 2020, VS cases were reported in NM from April to May, although total black fly abundance remained high until September. We investigated the hypothesis that transience of local VSV transmission results from transient abundance of key, competent black fly species. Additionally, we investigated whether irrigation canals in southern NM support a different community of black flies than the main river. Lastly, to gain insight into the source of local black flies, in 2023 we collected black fly larvae prior to the release of water into the Rio Grande River channel.

**Methods:**

We randomly sub-sampled adult black flies collected along the Rio Grande during and after the 2020 VSV outbreak. We also collected black fly adults along the river in 2021 and 2022 and at southern NM farms and irrigation canals in 2022. Black fly larvae were collected from dams in the area in 2023. All collections were counted, and individual specimens were subjected to molecular barcoding for species identification.

**Results:**

DNA barcoding of adult black flies detected four species in 2020: *Simulium meridionale* (*N* = 158), *S. mediovittatum* (*N* = 83), *S. robynae* (*N* = 26) and *S. griseum/notatum* (*N* = 1). *Simulium robynae* was only detected during the VSV outbreak period, *S. meridionale* showed higher relative abundance, but lower absolute abundance, during the outbreak than post-outbreak period, and *S. mediovittatum* was rare during the outbreak period but predominated later in the summer. In 2022, relative abundance of black fly species did not differ significantly between the Rio Grande sites and farm and irrigation canals. Intriguingly, 63 larval black flies comprised 56% *Simulium vittatum*, 43% *S. argus* and 1% *S. encisoi* species that were either extremely rare or not detected in previous adult collections.

**Conclusions:**

Our results suggest that *S. robynae* and *S. meridionale* could be shaping patterns of VSV transmission in southern NM. Thus, field studies of the source of these species as well as vector competence studies are warranted.

**Graphical Abstract:**

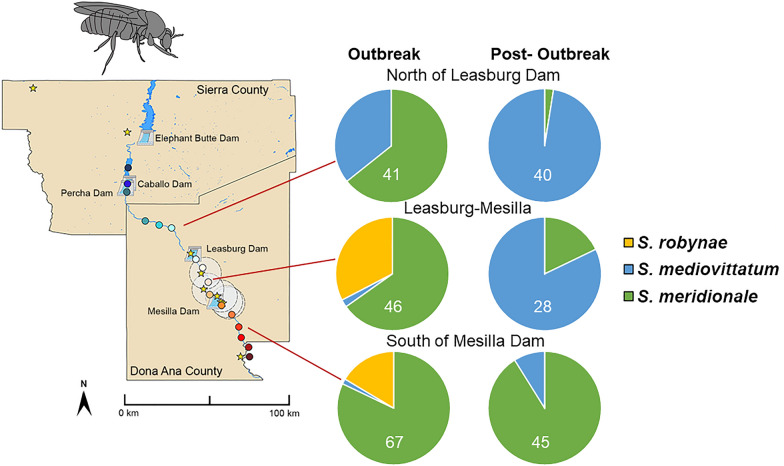

**Supplementary Information:**

The online version contains supplementary material available at 10.1186/s13071-024-06127-6.

## Background

Vesicular stomatitis virus (VSV) is a vector-borne, single-stranded, negative-sense RNA virus (family Rhabdoviridae; genus *Vesiculovirus*) comprising two serotypes: New Jersey (VSNJV) and Indiana (VSIV) [1, 2]. Clinical manifestations of vesicular stomatitis (VS) in hoofed domestic livestock (e.g. horses, cattle, pigs) include vesicular lesions in the mouth and on the feet or udders [3]. These lesions can cause lameness, reduced milk production and weight loss [4]. Outbreaks of VSV lead to substantial economic losses, caused by animal quarantines and decreased productivity [5]. Both VSV serotypes circulate endemically from northern South America to southern Mexico and emerge into the southern border states of the US at 3- to 10-year intervals, often expanding northward over the subsequent 2 to 3 years [6–11].

Several lines of evidence implicate black flies (*Simuliidae*) as important vectors of VSV transmission in the western US. First, during VSV incursions into the US, the path of virus migration usually follows rivers [3, 6, 7, 11–15]. While surface water per se is critical for most VSV vectors, black flies require running water for development [16]. Second, black fly species can disperse up to 500 km from their natal habitat [16], providing a potential mechanism for VSV expansion northwards. Third, vector competence studies have identified two North American black fly species as competent vectors for both serotypes of VSV: *Simulium vittatum* and *S. notatum* [17–20].

**Fig. 1:**
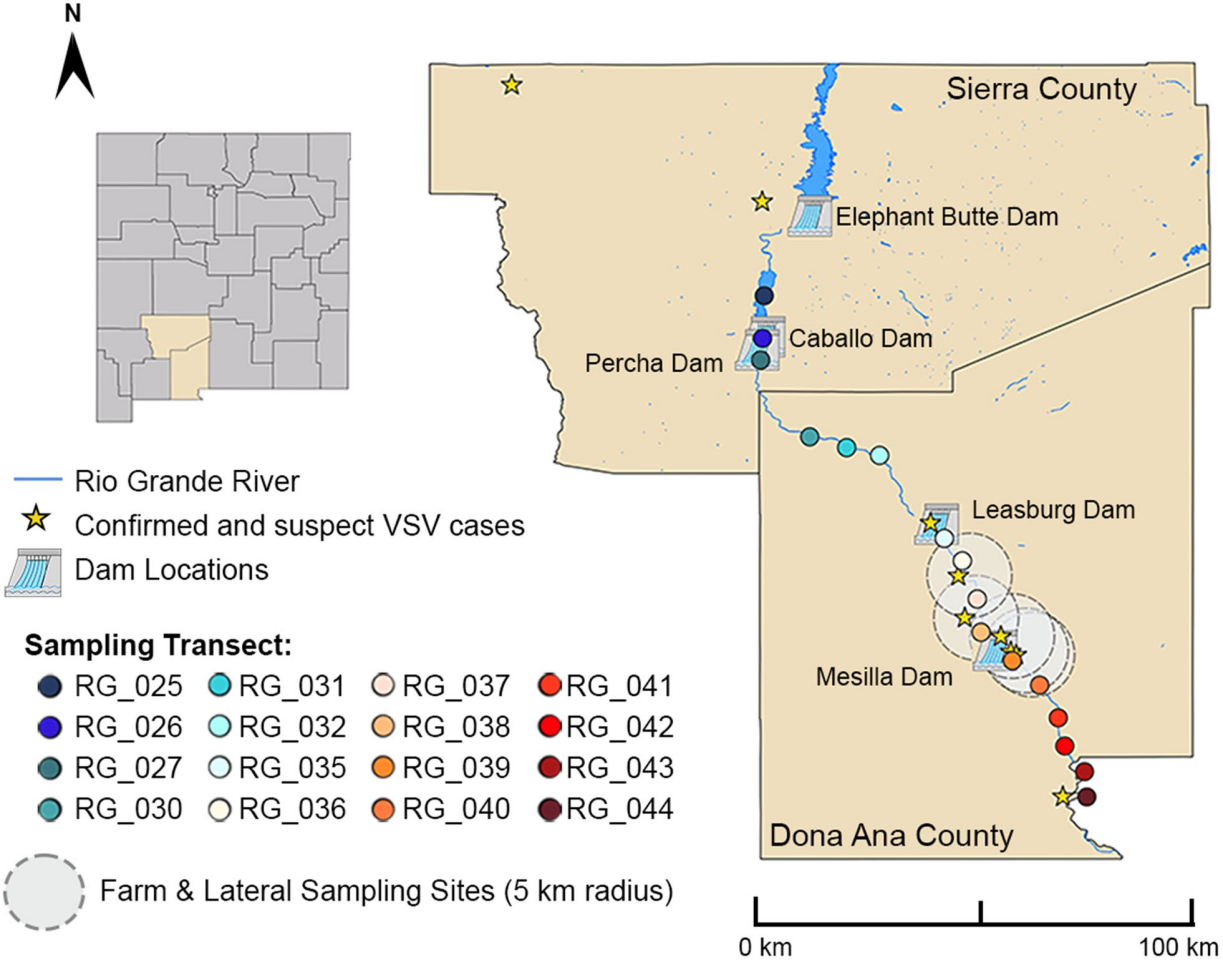
Doña Ana and Sierra counties in New Mexico showing the location sampling sites used in 2020, 2021, 2022 and 2023, as well as VS cases in 2020. Map adapted from Young, et.al [22]

**Fig. 2:**
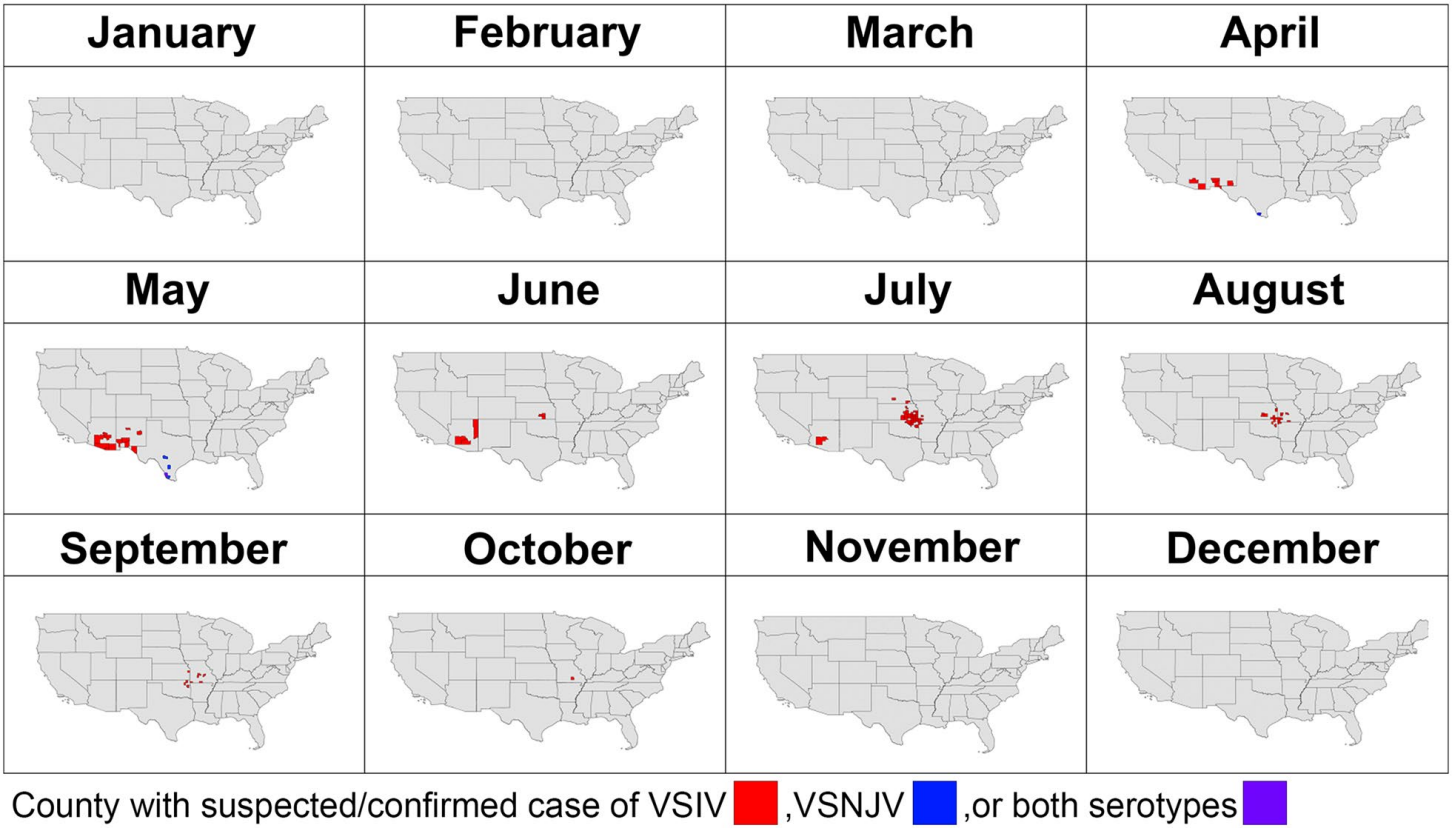
Vesicular stomatitis cases detected in individual counties (colored red) across the US in 2020. Data from [23]

Due to its proximity to Mexico, New Mexico (NM) has frequently been the entry point for VSV incursions into the US [21]. Thus, in 2020, we initiated surveillance of black flies along the Rio Grande. This river bisects the state and is the largest river and primary source of surface water in NM. Due to COVID-19 restrictions, collections were limited to southern NM, spanning Doña Ana and Sierra counties (Fig. 1) [22]. Serendipitously, in 2020, VSIV was detected in NM, with the index case reported in Doña Ana County on April 13, 2020. Cases were detected in NM through May 2020 (Fig. 2) [23]. The brevity of the 2020 VSIV outbreak in NM is notable, given that black fly abundance remained high in the region through September. This transience of local clinical disease reports is consistent with other locations in the US during the 2020 VSIV outbreak (Fig. 2).

We have considered several hypotheses for the brevity of local VSV circulation. First, herd immunity could truncate transmission. However, it seems unlikely that herd immunity could be achieved in the region over 2 months of transmission given the large number of potential hosts available. Moreover, it is not clear that herd immunity against VSV is ever possible, as hosts can become reinfected with the same strain of VSV within a brief period of time [4, 24–26]. Second, VSV could wane because total black fly abundance decreases, but black fly abundance remained high in southern NM through October 2020, long after VS cases had disappeared in the state. Third, vector competence of individual black fly species could decline in response to environmental changes. To our knowledge, the sensitivity of black fly vector competence for VSV to temperature has never been tested, although an association seems likely as temperature has been shown to affect black fly competence for other pathogens such as *Onchocerca volvulus* [27], and the competence of *Culicoides* midges for VSV is known to be temperature-dependent [28, 29]. Finally, VSV transmission may end when the abundance of competent black fly species wanes.

**Fig. 3:**
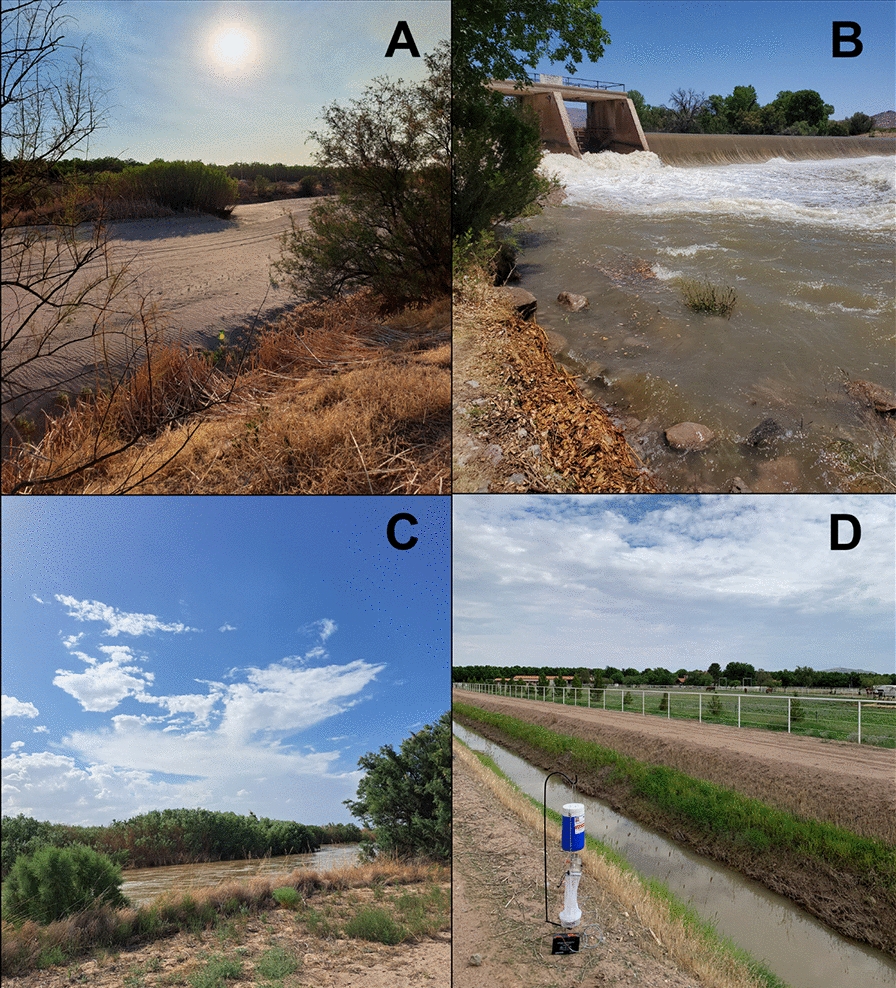
**A** Rio Grande River during winter months when flow ceases, **B** Percha Diversion Dam, **C** Rio Grande River when water is released (same vantage as panel **A**), **D** CDC light trap pictured next to irrigation canal in Southern NM

The current study investigated this last hypothesis by quantifying relative and absolute abundance of all black fly species collected during and after the 2020 VSV outbreak in NM. Previously, Young et al. [22] used molecular barcoding to identify black flies collected in the same region and time frame; however, these specimens were deliberately chosen based on morphological differences and do not offer an unbiased representation of species composition. As a nuance to this hypothesis, in 2022 we collected black flies at the Rio Grande itself as well as lateral irrigation canals and nearby farms to determine whether the black fly species compositions associated with irrigation canals differ from those in the main river channel. Finally, to identify potential sources of black flies in southern NM, where the river is dammed and runs dry during winter months (Fig. 3A, C), we collected black fly larvae from the surface of dams in 2023 prior to the release of water into the river channel.

## Methods

### Rio Grande River collection sites

Black fly collections in 2020 have been previously described [22]. Briefly, 16 transects spaced approximately 5 km apart were randomly selected spanning 149 km of the Rio Grande in Sierra and Doña Ana counties, NM. Each 1-km transect encompassed four trap collection sites spaced approximately 250 m apart, resulting in a total of 64 traps. This stretch of the river is interrupted by four dams (Fig. 1). Additionally, Percha Diversion Dam (Fig. 3B) sits about 5 km south of Caballo Dam. South of the Caballo Dam, the Rio Grande runs dry during winter months, while sites north of this dam have more consistent, albeit minimal, running water. To compare species composition during and after the 2020 outbreak period (April–June; July–November), the sampling region was divided into three sectors based on major dams: (i) north of Leasburg Dam, (ii) between Leasburg and Mesilla Dams and (iii) south of Mesilla Dam.

### Farm and irrigation canal collection sites

To investigate black fly abundance and species composition at farms, we first identified five farms with reported VSV cases in 2020. To maintain the anonymity of these farms, the four closest properties with livestock were also identified, and all five farms in a block were randomly ordered 1–5. All five properties were then visited in that order to seek permission to conduct vector sampling on the property; the first property to sign a permission form was chosen as the sampling site. We obtained permission from five properties ≤ 0.5 km from the five 2020 VSV outbreak locations. The five selected farms spanned 14.7 miles of Doña Ana County (3815 mi^2^). Three of the five farms held only horses, while the other two held horses and cattle. None of the farms sampled were directly adjacent to the Rio Grande.

Much of the agricultural land in the study region receives water diverted from the Rio Grande via irrigation canals (also known as laterals, ditches, acequias, main canals or conveyance canals). We identified the closest irrigation canal to each farm. The irrigation canals selected are primarily dirt reservoirs, except for occasional cement-lined areas near water gauges (Fig. 3D). The land cover around irrigation canals varied from bare dirt to heavy vegetation (orchards). The mean distance from farms to the closest irrigation canal was 529 m (minimum 169 m; maximum 925 m). In 2022, water was released into the Rio Grande in southern NM from June 6 to August 21, and irrigation canals only contained running water from June 6 to July 21.

### Adult black fly collections along the Rio Grande

Black fly collections along the Rio Grande were conducted using either Centers for Disease Control (CDC) light traps or Encephalitis Vector Surveillance (EVS) traps (BioQuip, California, USA), which were previously shown to be equally efficient for black fly capture [22]. Black flies were identified following Adler 2004 [16], separated and counted on a chill table (BioQuip, Compton, CA, USA). When traps contained > 2500 specimens, total counts were estimated by distributing the catch evenly across a gridded plate, counting a subset of grid cells and multiplying the mean number per grid cells × the total number of grid cells. Between April and November 2020, each site, excluding site RG_038, was sampled five times. RG_038 was sampled biweekly between March and November. Two breaks in sampling occurred in 2020 from June 6th to July 29th and from September 12th to October 8th. In 2021 and 2022, we continued bi-weekly sampling at site RG_038 throughout the year to monitor black fly abundance and resumed sampling other transects when the Rio Grande was flowing. In 2022, we continued longitudinal Rio Grande sampling using the sampling design above; however, we limited our efforts to sites RG_027, RG_031, RG_038 and RG_043 to focus resources on farm and irrigation canal sampling.

A subset of black flies collected in 2020 was randomly selected from two sampling seasons: the outbreak season spanned April 1–June 6, 2020, which is when VS cases were reported in NM and the post-outbreak season which spanned July 29–November 7, 2020. Additionally, we selected samples based on sector (designated by position of dams) for downstream analyses as follows. We sampled a total of six transects (24 traps) in the sector north of the Leasburg Dam, four transects (16 traps) between Leasburg and Mesilla Dams and six transects (24 traps) south of the Mesilla Dam. From the selected traps, 5% of the total number of collected black flies was randomly selected for DNA extraction, except for traps with > 200 black flies, from which 10 black flies were selected, and traps with < 20 black flies, from which 1 black fly was selected. This sampling scheme resulted in 298 black flies from 82 traps selected for barcoding across three sectors and two seasons.

### Adult black fly collections at farms and along irrigation canals

**Fig. 4:**
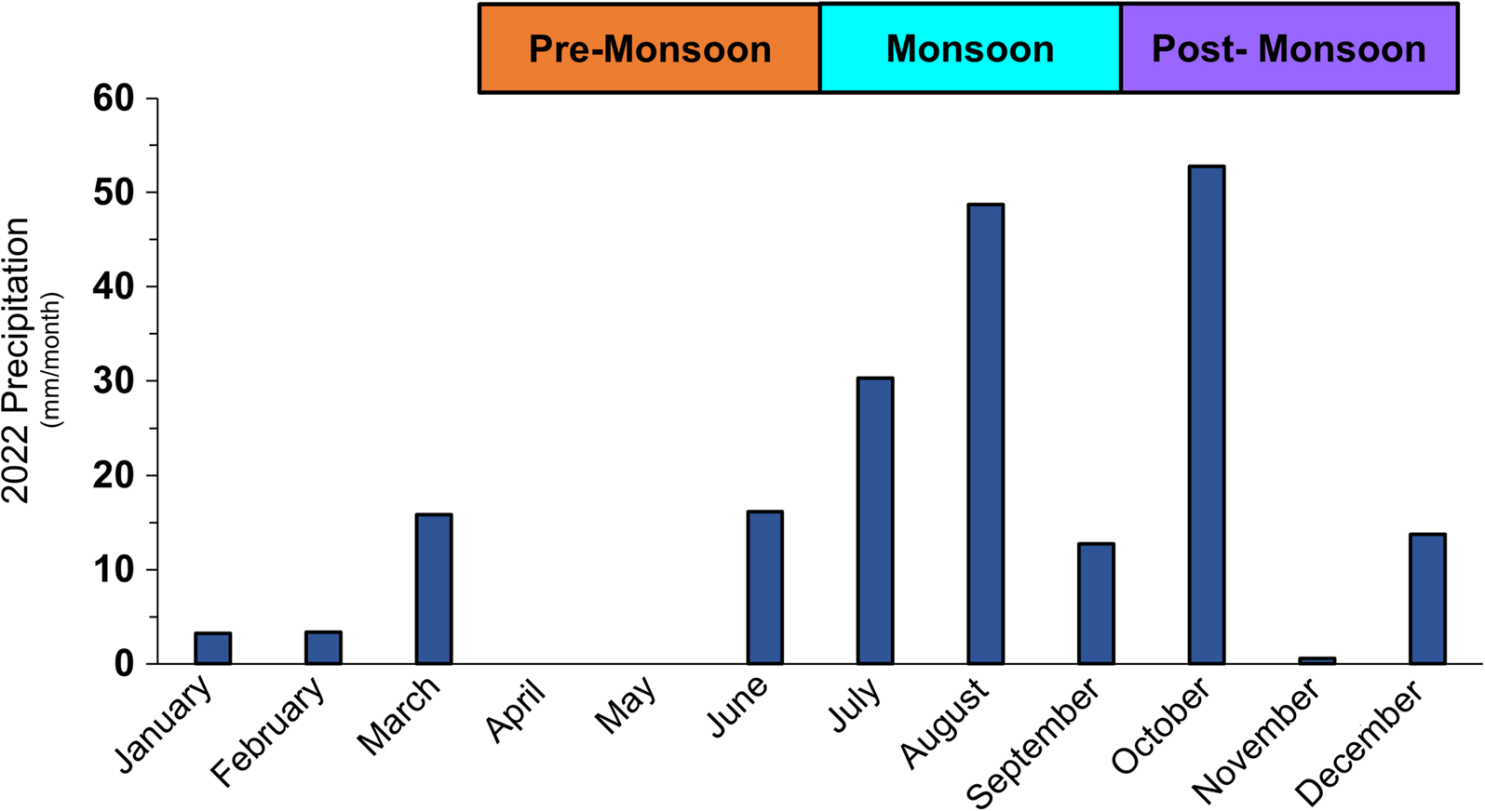
Precipitation by month and rainfall period (pre-monsoon (6/6/22–7/6/22); mid-monsoon (7/21/22–9/16/22); post-monsoon (9/30/22–12/6/22) at site RG_038 in Southern New Mexico, which is consistently sampled year-round

Three traps were placed at each farm and each irrigation canal. Traps within farms were set in a triangular configuration at varying distances because of varying property sizes (minimum distance between traps was 40 m). Traps along irrigation canals were set approximately 250 m apart. Three sampling periods were chosen based on patterns of monsoonal rainfall, which lasts from June 15 to September 30 in southern NM: pre-monsoon, mid-monsoon and post-monsoon (Fig. 4). Traps were placed between 0700 and 1200 h and ran for 3 consecutive days. Dry ice, catch bags and batteries were replaced daily.

### Larval black fly collections along the Rio Grande

In 2023, black fly larva collections were conducted between May 1 to May 5, prior to the release of water into the Rio Grande from Caballo Dam on May 13. Larval surveillance was conducted at a subset of adult black fly collection sites (RG_027, 030–032 and 035–041) but all were completely dry, and no larvae were detected. Larval surveillance was also conducted on the surfaces and immediate vicinity of Mesilla Dam, Leasburg Dam, Percha Dam and Caballo Dam (RG_026) (Figs. 1, 3B). Larvae were detected on the latter two dams, where a small volume of overflow water continued to run, as well as rocks located in a small stream (0.5–2.0 m wide) near Caballo Dam. Larvae were collected by gentle scraping into a fine-gauge net. Larvae were placed into tubes and held in a cooler with ice until they were transferred into the − 80 °C freezer.

### CoxI barcoding for species identification

DNA extractions were conducted using either TRIzol LS reagent (Invitrogen, Waltham, MA, USA) protocol following Young et al. [22] (*N* = 292) or the Zymo Quick-DNA Tissue/Insect kit (*N* = 176). For the latter approach, black fly samples were homogenized in 750 μl of Bashing Bead Buffer (Zymo Research, Irvine, CA, USA) using the Qiagen TissueLyser II Bead Mill Sample Disruption Preparation (Qiagen, Hilden Germany) at a frequency of 24.9 1/s for 15 min. DNA was then isolated using the Zymo Quick-DNA Tissue/Insect Microprep kit (Zymo Research, Irvine, CA, USA) following manufacturer’s instructions save that, rather than adding beta-mercaptoethanol to the entire stock of Zymo genomic lysis buffer, fresh beta-mercaptoethanol was added to small aliquots of lysis buffer each time an extraction was carried out. DNA was eluted using 25 μl of provided elution buffer, and DNA concentrations were determined using a Nanodrop spectrophotometer (Thermo-Fisher Scientific, Waltham, MA, USA).

Amplicons were produced using sample DNA (100 ng) and universal Cox1 primers designed by Folmer [30]: LCO1490: 5′GGTCAACAAATCATAAAG ATATTGG-3′ (forward) and HCO2198: 5′-TAAACTTCAGGGTGACCAAAAAATCA-3’(reverse). PCR cycling conditions were 96 °C for 1 min, 35 times: 94 °C for 1 min, 52 °C for 1 min, 72 °C for 1.5 min, and reactions were held at 8 °C at completion. DNA amplicons were visualized using gel electrophoresis on 1% agarose gel with 1X SYBR safe DNA gel stain (Thermo-Fisher Scientific, Waltham, MA, USA). PCR product (10 μl) was mixed with 1 μl of 10X BlueJuice Gel Loading Buffer (Thermo-Fisher Scientific, Waltham, MA, USA) and loaded into wells. Gels were run at 100 V for 1 h and then visualized using the GelDoc Go Imaging System (Bio-Rad, Hercules, CA, USA). If the sample was successfully amplified, the remaining 15 μl of PCR product was purified using the Roche High Pure PCR Product Purification Kit (Sigma-Aldrich, St. Louis, MO) following the manufacturer’s instructions and sent to the University of Texas at El Paso genomics core laboratory for sequencing (3500 Genetic Analyzer; Applied Biosystems, Waltham, MA). Sequences were analyzed and cleaned in Geneious Prime version 2021.1.1 (https://www.geneious.com). Cleaned amplicons, generally 633 bp in length, were compared to sequences in NCBI database and BOLD (https://v4.boldsystems.org/), as well as 11 CoxI barcodes generated from morphologically confirmed *Simulium* species likely to be found along the Rio Grande including *Simulium argus, S. bivittatum, S. encisoi, S. griseum/S. notatum, S. mediovittatum, S. meridionale, S. paynei, S. robynae, S. trivittatum* and * S. vittatum* [22].

Sample sequences longer than 500 bp, voucher specimen sequences and an outgroup sequence (*Liohippelates pusio*, Genbank Accession Number: HQ945300, accessed on 4 June 2021) were aligned using the MAFFT aligner [31] in Geneious 2023.1 (Biomatters Ltd., Auckland, New Zealand). The alignment of the barcoding region (without stop codons and gaps) was used for phylogenetic construction to determine species identities based on relationships to voucher specimen sequences [22]. ModelTest-NG was used to determine the best evolutionary model [32, 33]. The best fit maximum likelihood tree was inferred with RaxML version 8. Rapid bootstrapping was also conducted with 1000 bootstrap replicates.

Samples that produced no or weak amplicons were re-run using the same PCR conditions noted above but with the previous PCR product as the template. Samples that still produced no amplicons were removed from analysis.

### Environmental data

Following Young et al. [22], we analyzed the association of four environmental variables with black fly abundance: Rio Grande flow rates, air temperature, precipitation and normalized difference vegetation index (NDVI), a measure of vegetation greenness. River flow rates were obtained from the Elephant Butte Irrigation District (https://www.ebid-nm.org/scada, accessed 16 February 2023). The nearest upstream river gauge was used to determine daily mean flow rate (ft^3^/s). Maximum and minimum daily temperatures for 2020–2022 sampling dates were obtained using Daymet in R (https://daymet.ornl.gov/, accessed on 4 March 2022). Precipitation data (monthly total) obtained from Daymet were used to calculate 2-month and 1-year lagged precipitation data for each sampling site (total precipitation 2 months/1 year prior to sample collection month). NDVI measurements (30 m resolution) were obtained using Google Earth Engine from the Landsat 8 Collection 1 (8-Day composite) dataset (2020 and 2021; courtesy of the US Geological Survey). Since Landsat 8 discontinued releasing data on Google Earth Engine, 2022 NDVI values were calculated from Landsat 8 Collection 1 (8-day composite) dataset using USGI EarthExplorer (courtesy of the US Geological Survey) and qGIS Desktop 3.28.2 (QGIS Development Team (2023), “http://qgis.osgeo.org”). NDVI layers were combined in Google Earth Engine to generate shapefiles containing bi-monthly NDVI mean values. Site-specific NDVI values were then extracted in ArcMap 10.6.1 (ESRI, CA, USA).

### Statistics

Contingency table analysis was used to compare relative abundance of individual species between outbreak and post-outbreak periods and among the three sampling sectors. Data were transformed by adding 1 to the total number of each species count at each river section to avoid zero values.

To test for differences in black fly abundance across seasons, the mean number of black flies sampled per day was calculated for each transect. A log_10_ + 1 transformation was used to render these values normally distributed. A one-way ANOVA was run for each season (spring, summer or fall), and the Tukey-Kramer Honest Significant Difference (HSD) test was used to identify statistically significant pairwise differences. A two-factor (month and year) ANOVA was used to compare environmental variables among years. A generalized linear model (GLM) with normal distribution and maximum likelihood estimation was used to test associations in the absolute abundance of individual species per trap with environmental variables or total black fly abundance. All analyses were run in JMP version 16.0 (SAS Institute Inc., Cary, NC, USA). Shannon Diversity Index was calculated using the "vegan" package in R [34], and Shannon Equitability Index (H) was calculated by dividing Shannon Diversity Index (H) values by the natural log of number of unique species.

## Results

All raw data for adult and larval black fly collections are presented in Additional file 1.

### Black fly identity and relative abundance during and after the 2020 VSV outbreak

**Fig. 5:**
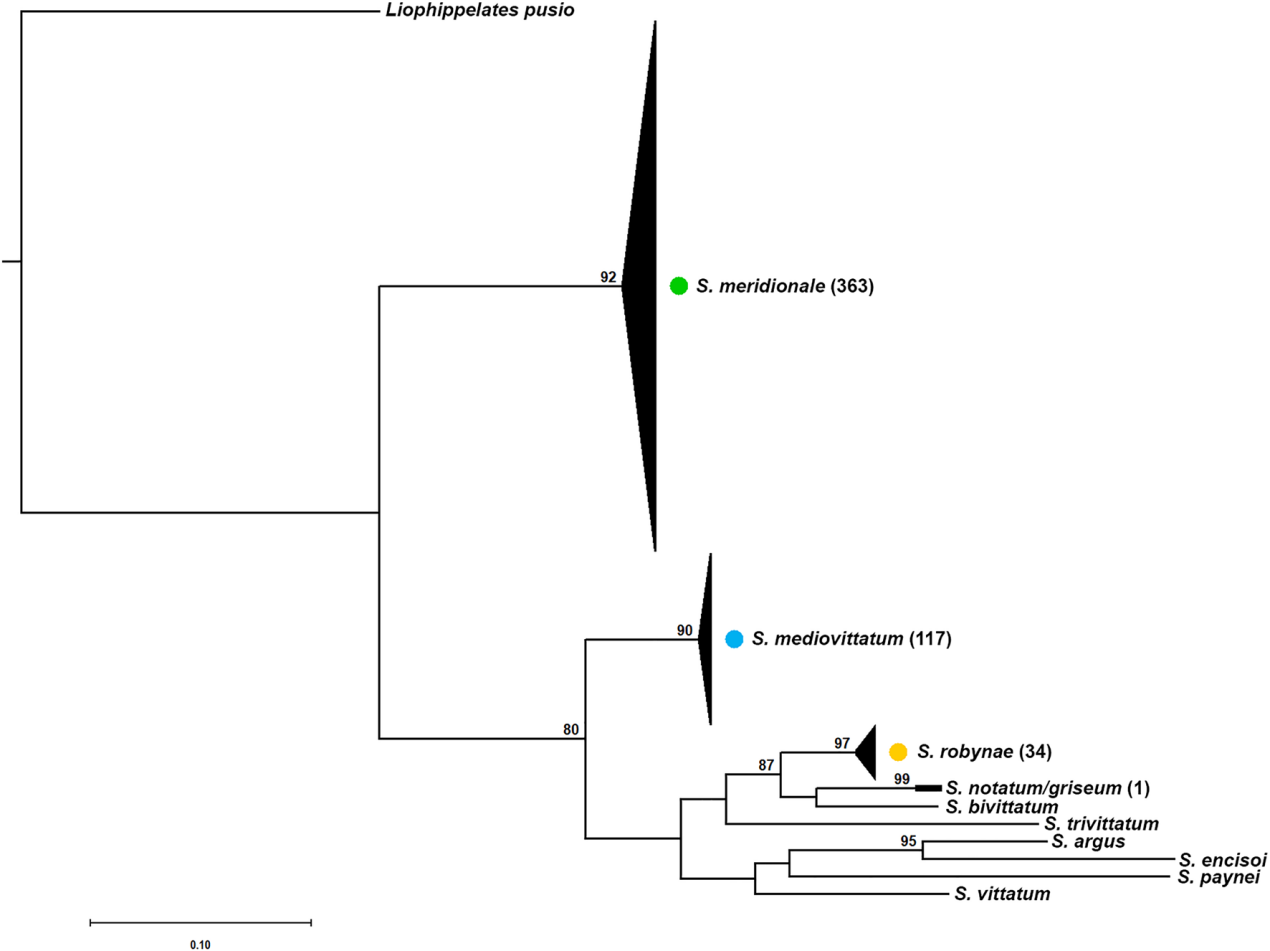
Maximum likelihood phylogeny [RaxML v.8, bootstrap values > 75 (1000 replicates) are noted at the nodes] of all adult black fly samples successfully barcoded in NM from 2020–2022, 11 voucher specimen sequences and one outgroup, *Liohippelates pusio*, inferred from mtDNA CoxI. The numbers of total barcodes (excluding voucher sequence) for each *Simulium* species are listed next to the taxon name, but because of the high number of barcodes every individual was not listed in the phylogeny

Of 298 individual black fly samples from 2020 selected for barcoding, 267 (91%) produced CoxI barcodes, comprising *S. meridionale* (*N* = 158)*, S. mediovittatum* (*N* = 82)*, S. robynae* (*N* = 26) and *S. griseum/notatum* (*N* = 1) [Shannon Diversity Index (*H*) = 0.92; Shannon Equitability Index (*E*_*H*_) = 0.66]. Black fly samples within the same species had an average of 98.4% identity to each other and an average of 97.3% identity to voucher CoxI sequences (Additional files 2, 3). Additionally, phylogenetic analysis demonstrated that CoxI sequences grouped to voucher sequences with high bootstrap support (Fig. 5).

Barcodes from black flies collected during the outbreak period (*N* = 155) comprised four species: *S. meridionale* (*N* = 111)*, S. mediovittatum* (*N* = 17)*, S. robynae* (*N* = 26) and *S. griseum/notatum* (*N* = 1) (*H* = 0.81; *E*_*H*_ = 0.59). *Simulium griseum/notatum* was excluded from statistical analysis because of small sample size. Barcodes from black flies collected during the post-outbreak period (*N* = 113) comprised two species: *S. mediovittatum* (*N* = 66) and *S. meridionale* (*N* = 47) (*H* = 0.68; *E*_*H*_ = 0.98) (Fig. 5).

**Fig. 6:**
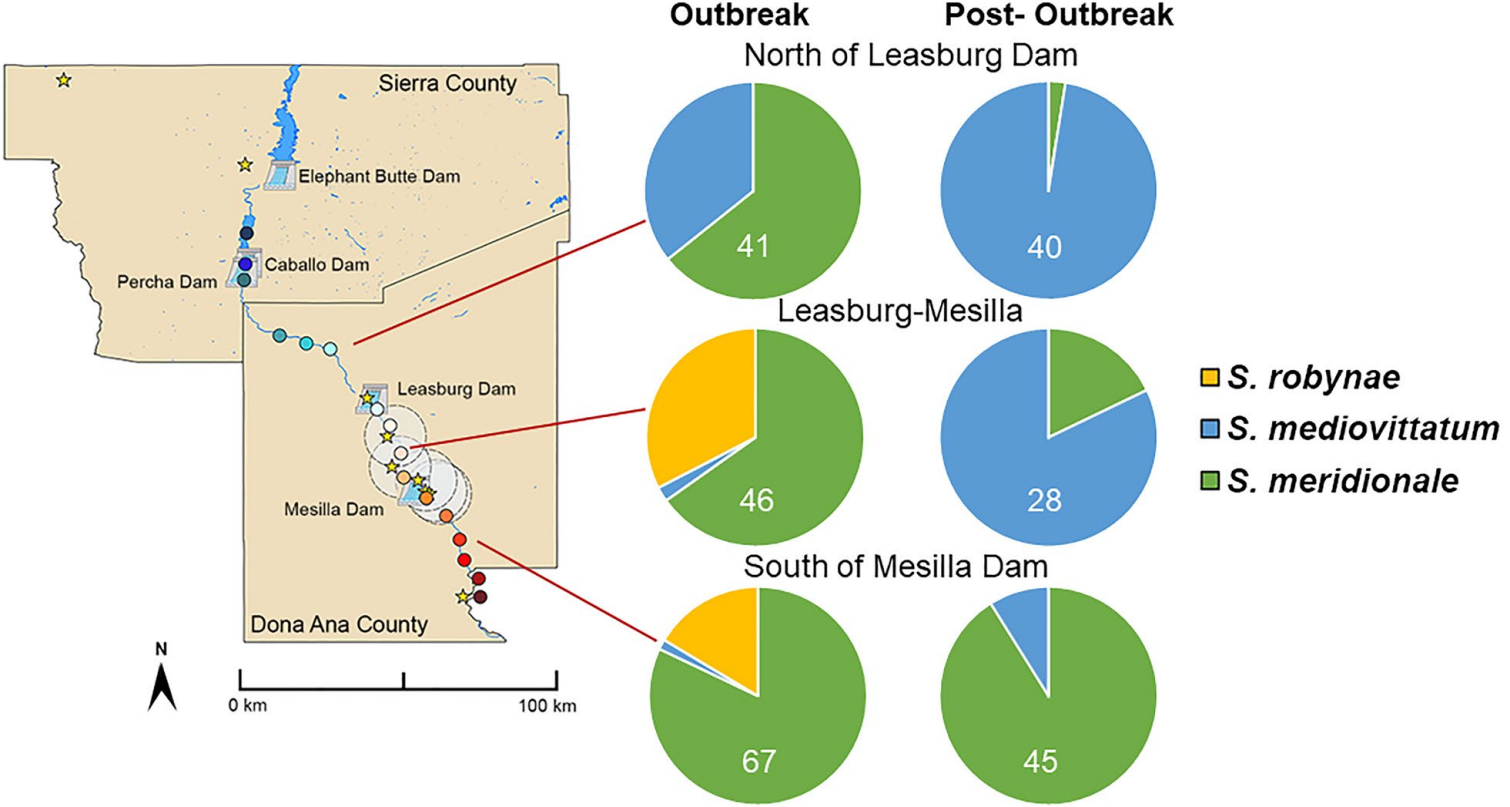
Relative black fly species abundance during the 2020 VSV outbreak and post-outbreak period within different sectors of the Rio Grande

In each of the three sectors of the river analyzed, relative species abundance differed significantly between the outbreak and post-outbreak period (df = 2, *χ*^2^ = 70.09, *P* < 0.0001) (Fig. 6). *Simulium robynae* was only detected during the outbreak period. *Simulium meridionale* predominated during the outbreak period, and *S. mediovittatum* predominated post-outbreak north of the Leasburg Dam (df = 2, *χ*^2^ = 34.2, *P* < 0.0001), between Leasburg and Mesilla Dam and south of the Mesilla Dam (df = 2, *χ*^2^ = 9.2, *P* = 0.0099).

Additionally, relative abundance of black fly species varied significantly among sampling sectors (Fig. 6). During the outbreak period, relative abundance of *S. robynae* was significantly higher and relative abundance of *S. mediovittatum* was significantly lower downstream of the Caballo Dam, where many VS cases were detected in 2020 (df = 4, *χ*^2^ = 40.4, *P* < 0.0001). After the outbreak, *S. mediovittatum* predominated at traps north of the Mesilla Dam (df = 4, *χ*^2^ = 87.4, *P* < 0.0001).

### Absolute abundance of black fly species in 2020

**Fig. 7:**
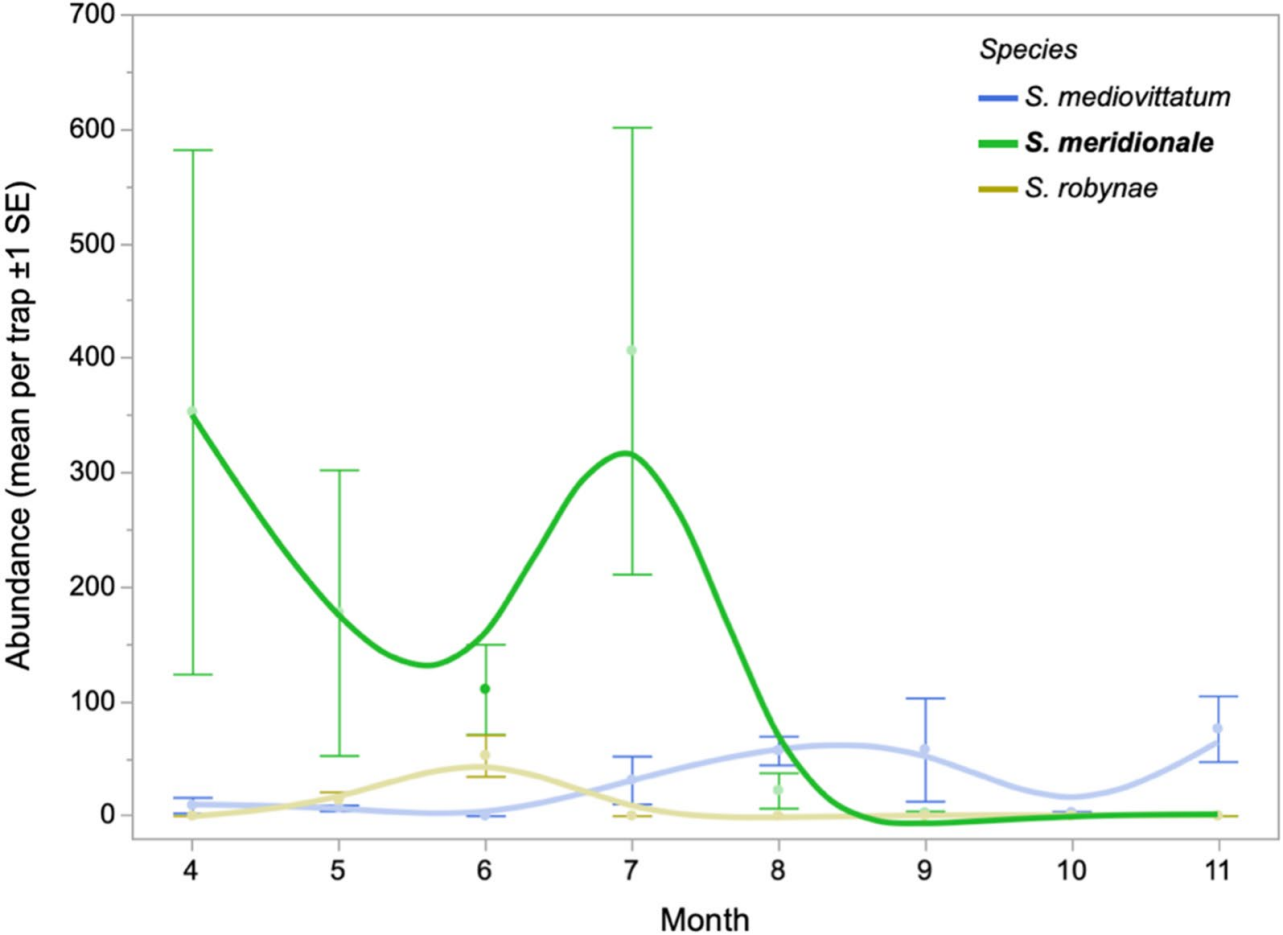
Absolute abundance of black fly species across months in 2020. Note that the X axis starts in April (Month 4) and ends in November (Month 11)

Absolute abundance of the three *Simulium* species detected during this study was calculated by multiplying the percentage of each species detected in the trap by the total number of black flies in the trap. In 2020, absolute abundance of *S. mediovittatum* remained low until August. *Simulium meridionale* had the highest absolute abundance through August, with a peak in July, while absolute abundance of *S. robynae* remained low, and the species ceased to be detected after June (Fig. 7).

### Total black fly abundance on the Rio Grande: 2020–2022

**Fig. 8:**
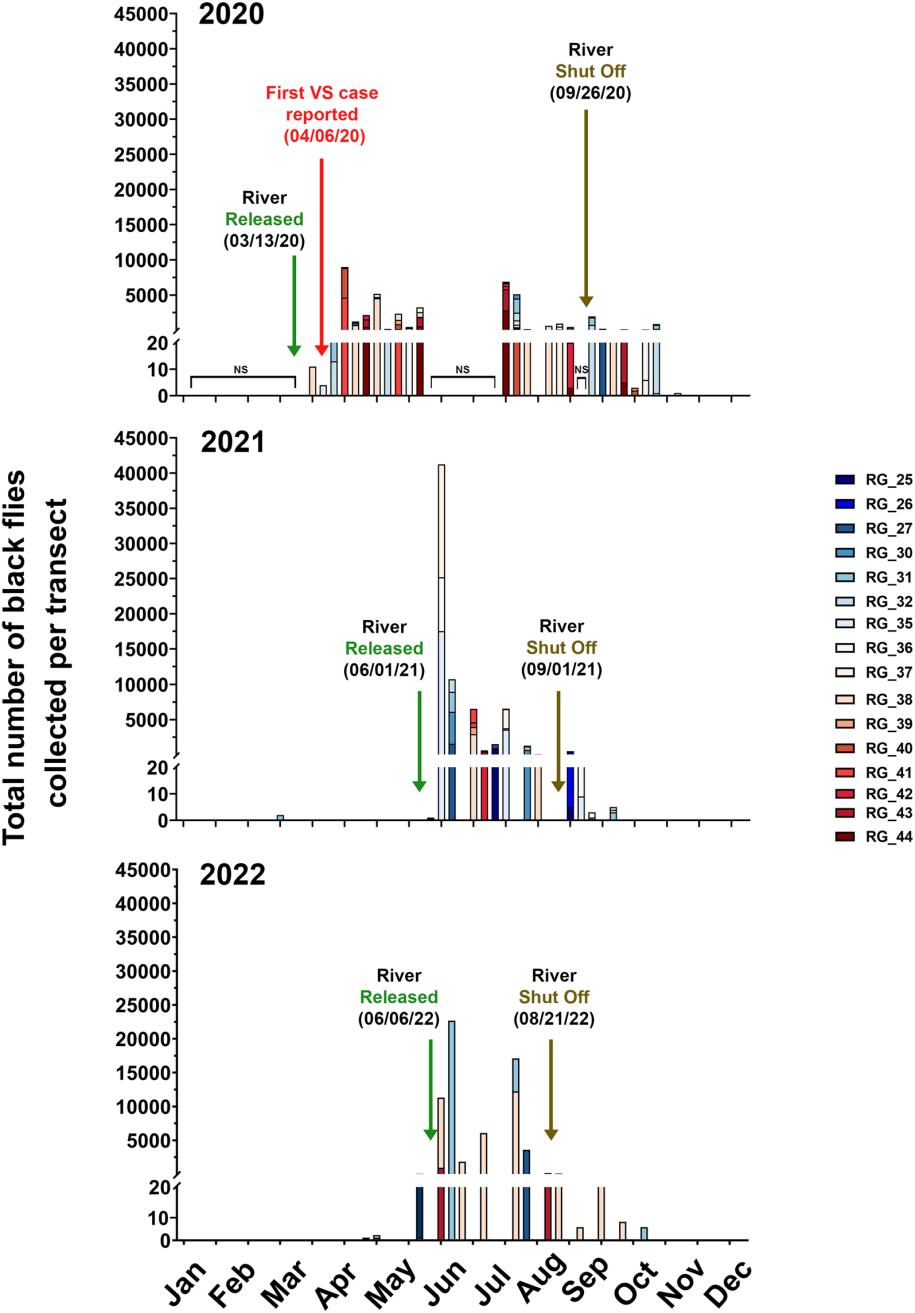
Total number of black flies collected in 2020 (2020 data from Young et al. 2021), 2021 and 2022

In 2020, as previously reported [22], black fly abundance spiked following the release of the river from the Caballo Dam on March 13, 2020, and remained high until the river shut-off on September 26, 2020 (Fig. 8). In 2021, the Rio Grande was released on June 1 and shut off on September 1. As in 2020, black fly abundance peaked a few weeks after the release of the river and decreased gradually thereafter (Fig. 8). In 2022, the Rio Grande was released on June 6 and shut off on August 21. As expected, black fly abundance spiked following river release and waned after the flow was shut off (Fig. 8). Despite shorter durations of river flow in 2021 and 2022, and less sampling effort in 2022, the total number of black flies collected increased after 2020 (2020: *N* = 40,699; 2021: 77,972; 2022: 62,985). No VSV cases were reported in the US in 2021 or 2022.

### Black fly abundance at farms and irrigation canals

**Fig. 9:**
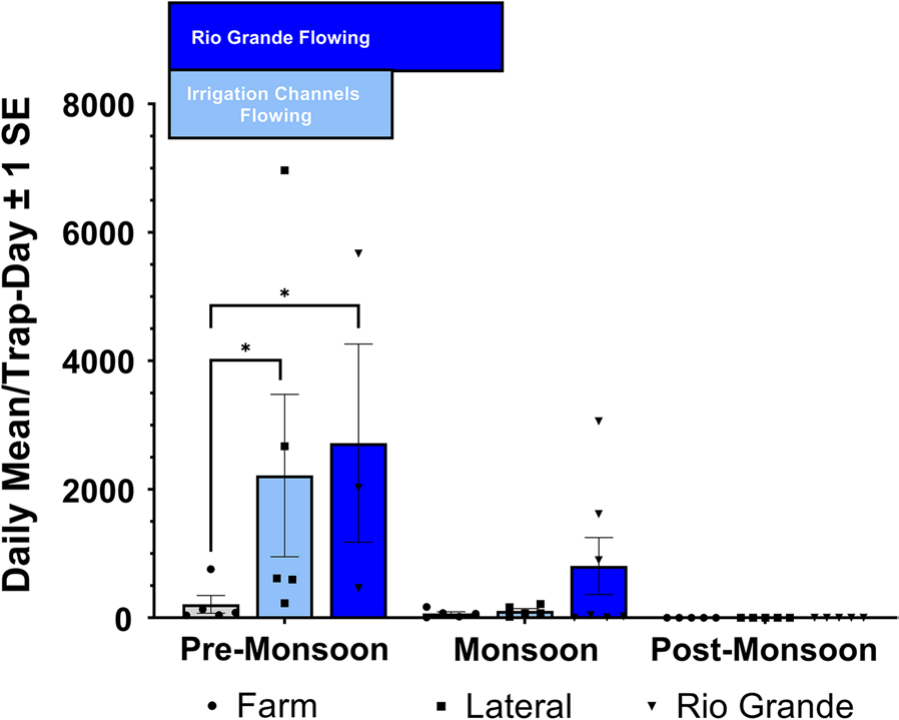
Average number of black flies collected per transect per day at irrigation canals, farms and Rio Grande sites across the three sampling seasons (pre-monsoon, mid-monsoon, post-monsoon). Abundance was only compared among land cover types during the pre-monsoon period

Black fly abundance at farm and irrigation canals in 2022 was highest during the pre-monsoon period, which corresponds to the release of the Rio Grande on June 6 (Fig. 9). Despite heavier rainfall during the mid-monsoon period, black fly numbers decreased; it was during this period that the river flow was shut off. Very few black flies were collected during the post-monsoon period. The same trend in abundance was observed at sites along the Rio Grande, with abundance peaking during the pre-monsoon period and gradually decreasing thereafter (Fig. 9). To ensure a fair comparison, we limited comparison of black fly abundance to the pre-monsoon period when both the river and irrigation canals were flowing. During this period, black fly abundance differed among the three types of sites sampled [Rio Grande, farm and irrigation canal (ANOVA, *F*_(2,10)_ = 6.71, *P* = 0.01], with abundance significantly lower at farm sites than Rio Grande sites (Tukey-Kramer HSD, *P* = 0.02) and irrigation canal sites (Tukey-Kramer HSD, *P* = 0.04) but no significant difference between Rio Grande and irrigation canal sites.

### Association of environmental variables with absolute black fly abundance

Following Young et al. [22], the association of normalized total black fly abundance (site total divided by mean number of flies sampled across all sites in the same month of sampling) with four environmental variables, namely river flow rate (feet^3^/s), maximum temperature (^o^C, day sampled), NDVI (mean; bi-monthly) and rainfall (mm), with rainfall being partitioned into three classes (during the month of sampling, the previous 2 months and the previous year), was analyzed. To avoid confounding seasonal effects, this analysis was conducted separately for collections in the spring (March–May), summer (June–August) and fall (September–November). Data from 2021 and 2022 were analyzed similarly; however, because the Rio Grande was not released until June 6, these analyses were limited to summer and fall. In 2021 and 2022, we detected no significant associations between normalized black fly abundance and environmental variables in any season. To investigate why associations were detected in 2020 but not in the latter 2 years, we tested for differences among years in each variable. We detected a significant interaction between year and month for precipitation at each site [two-factor ANOVA, (ANOVA, *F*_(20, 315)_ = 52.1, *P* = 0 < 0.0001]; in general, precipitation in summer was heavier in 2021 and 2022 than in 2020, and precipitation in fall was heavier in 2022 than in the other 2 years. Similarly significant interactions between month and year were detected for maximum and mean temperature and river flow rate. The year 2020 was dramatically hotter than 2021 or 2022 in August. River flow rate was equivalent among the three years only in June and was otherwise substantially higher in 2020. Finally, a two-factor ANOVA showed a significant difference among years, with no interaction effect with month, in bimonthly NDVI [ANOVA, *F*_(8, 135)_ = 5.56, *P* < 0.005]; NDVI was highest in 2021, slightly lower in 2020 and substantially lower in 2022.

Association of the absolute abundance of individual species collected in 2020 with key environmental variables was also analyzed. In spring collections, 100 barcodes were amplified for four species: *S. mediovittatum* (*N* = 13), *S. meridionale* (*N* = 76), *S. robynae* (*N* = 10) and *S. griseum/notatum* (*N* = 1) (*H* = 0.75; *E*_*H*_ = 0.54); these samples originated from a total of 29 traps containing 7583 black flies. Generalized linear models were used to determine whether absolute species abundance was associated with any environmental variables. To mitigate impacts of multiple comparisons, and because percentage samples lack independence, a more stringent cutoff of alpha = 0.01 was used to test significance. A positive association between absolute abundance of *S. meridionale* and 2-month lagged precipitation was the only significant association detected (GLM; df = 1, *χ*^2^ = 9.95, *P* = 0.0016) (Table 1).

**Table 1:** Environmental variables associated with absolute abundance of *Simulium* species collected in 2020

Season	Total *N* in traps	Total black fly abundance	*S. robynae* abundance	*S. meridionale* abundance	*S. mediovittatum* abundance
Spring (March–May)	7571	(+) Precipitation (2 month lagged)	None significant	(+) Precipitation (2 month lagged)	None sampled
Summer (June–August)	5080	(+) Maximum temperature	(−) Precipitation (2 month lagged)	None significant	None significant
Fall (September–November)	912	(−) NDVI (+) Precipitation (1 year lagged)	None sampled	Not analyzed because of small sample size	Not analyzed because of small sample size

Factors analyzed: NDVI (bi-monthly mean), average flow rate (feet^3^/ s), maximum temperature (°C; daily), precipitation of month sampled, 2-month lagged precipitation and 1-year lagged precipitation (mm). Blue represents a positive association, and orange represents a negative association. Associations with total black fly abundance were previously reported by Young et al. [18]

For collections conducted in the summer, 116 barcodes were amplified for three species: *S. mediovittatum* (*N* = 31), *S. meridionale* (*N* = 69) and *S. robynae* (*N* = 16) (*H* = 0.94; *E*_*H*_ = 0.85); these samples originated from a total of 24 traps containing 5080 black flies. Absolute abundance of *S. robynae* was negatively associated with 2-month lagged precipitation (GLM, *df* = 1, *χ*^2^ = 7.24, *P* = 0.007) (Table 1).

For collections conducted in the fall, 39 barcodes were amplified for two species: *S. meridionale* (*N* = 4) and *S. mediovittatum* (*N* = 35) (*H* = 0.33; *E*_*H*_ = 0.48); these samples originated from a total of 21 traps containing 912 black flies. Given the small sample sizes, we did not conduct statistical analyses on these data.

### Black fly identity and relative abundance at farm, irrigation canal and Rio Grande sites in 2022

**Fig. 10:**
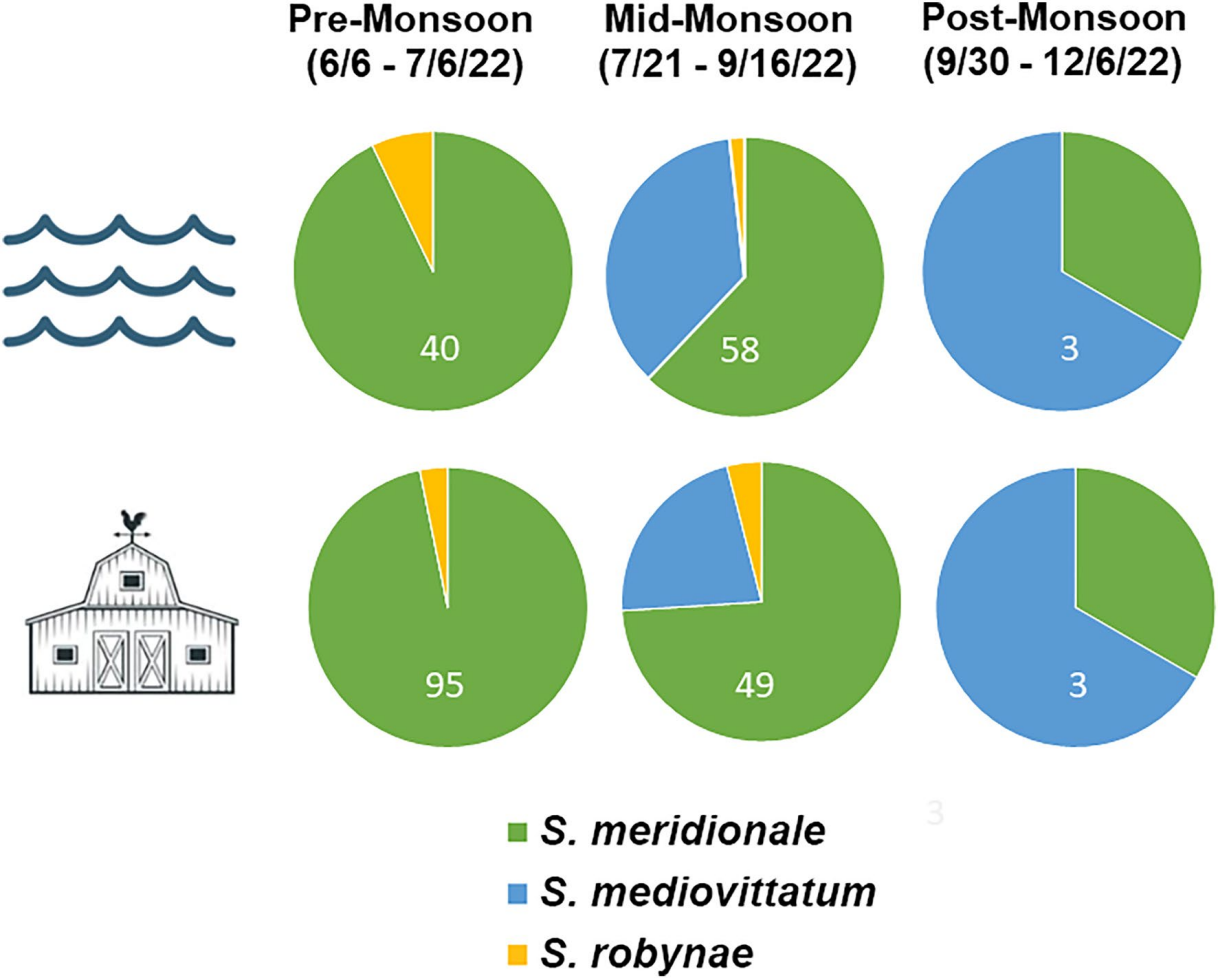
Relative abundance of black fly species in 2022 at farm and irrigation canal sites compared to Rio Grande sites. The top panel represents Rio Grande sites, and the lower panel represents farm sites

Of 170 black flies collected from farm and irrigation canal sites, 147 samples produced successful CoxI barcodes, while at Rio Grande sites, 101 of 112 individual black fly specimens produced barcodes (Fig. 10, Additional files 2, 3), including 44, 63 and 5 from the pre-, mid- and post-monsoon seasons, respectively. The same three species were detected across all sites: *S. meridionale* (*N* = 204), *S. mediovittatum* (*N* = 35) and *S. robynae* (*N* = 9) (*H* = 0.56; *E*_*H*_ = 0.51), and there were no significant differences in relative abundance among river, farm and lateral canal sites during the pre-monsoon (df = 2, *χ*^2^ = 1.93, *P* = 0.38) and monsoon (df = 2, *χ*^2^ = 3.18, *P* = 0.20) periods. Sample size was too small (*N* = 6) to analyze differences during the post-monsoon period (Fig. 10).

### Identity of black fly larvae collected prior to the release of the Rio Grande in 2023

Molecular barcoding of larvae revealed three species collected from two New Mexico dams: *Simulium vittatum* (*N* = 35), *S. argus* (*N* = 27) and *S. encisoi* (*N* = 1) (*H* = 0.76; *E*_*H*_ = 0.69) (Table 2, Additional files 2, 3, 4).

**Table 2:** *Simulium* species detected via molecular barcoding of larvae from Caballo and Percha Dams in May 2023 prior to the release of the Rio Grande

Dam	*Simulium* species	*N*	Average % identity to voucher specimen barcode
Caballo Dam	*S. vittatum*	27	99.4%
	*S. argus*	4	98.5%
	*S.encisoi*	1	99.8%
Percha Dam	*S. vittatum*	8	99.3%
	*S. argus*	23	98.9%
	*S.encisoi*	0	N/A

## Discussion

Our study investigated the hypothesis that VSV transmission in NM during the 2020 outbreak was curtailed because the abundance of critical, competent vector species declined. Using molecular barcoding, we identified four species, *S. meridionale, S. mediovittatum, S. robynae* and *S. griseum/notatum*, among randomly selected black flies collected during and after the 2020 VSV outbreak in southern NM. Reflecting the difference between deliberate and random sample selection, Young et al. [22] detected five different species in 99 specimens from the same collections, including multiple representatives of each, whereas we detected only four species of 267 specimens barcoded, one of which, *S. griseum/notatum*, was represented by a single sample.

In the current study, and consistent with Young et al. [22], *S. robynae* was only detected during the 2020 outbreak period, and only in the southern half of the sampling area. Absolute abundance of *S. robynae* was negatively associated with 2-month lagged precipitation. In 2020, VSIV was detected from *S. robynae* sampled in NM [22], implicating this species as a potential key vector for VSV transmission. Thus, the vector competence of this species should be experimentally assessed in future research. In the US, this species is typically found along the Texas and New Mexico border [16], and while* S. robynae* was first reported along the Rio Grande in 1993 [35], studies regarding its habitat and ecological preferences are limited. However, Adler reported that immature stages prefer shallow water flow [16], which could explain the presence of *S. robynae* in southern NM where river flow is lighter and more sporadic than in northern regions.

*Simulium meridionale*, also known as the turkey gnat, dominated black fly communities during the outbreak season and through July, particularly south of the Caballo Dam. A similar July decline of *S. meridionale* was observed in Mississippi [36]. The range of *S. meridionale* stretches across much of the US and into Mexico [16]. Larvae are typically found on loose debris and vegetation in swift, deep rivers with sandy bottoms and rocky bases [16]. During the outbreak period, absolute abundance of *S. meridionale* was positively associated with 2-month lagged precipitation, which could correspond to heavier river flows. In 2020, VSIV was detected from *S. meridionale* sampled in NM [22] as well as from *S. meridionale* in a VSIV infected farm in Kansas in 2020 [37]. *Simulium meridionale* can travel 30 km away from flowing water ways [16], potentially carrying VSV from waterways to farms. This, along with its broad host range [16] and high relative abundance during the 2020 VSV outbreak, could implicate *S. meridionale* as a VSV vector. However, *S. meridionale* remained abundant throughout the entire sampling season, and therefore its dynamics do not coincide with the cessation of VSV transmission in NM. Nonetheless, further study of the vector competence of *S. meridionale* for VSV is warranted.

*Simulium mediovittatum* predominated during the post-outbreak season and at traps north of the Caballo Dam. This species occurs from Texas southward into northern Mexico; it was first reported in NM in 2020 by Young et al. [22]. Larvae are commonly found in shallow flows of the Rio Grande drainage [16], which could explain why they are able to persist into the fall season when water flow diminishes. While *S. mediovittatum* tested positive for VSV in 2020 [22], its rarity during the outbreak period makes it a less likely candidate for maintaining VSV in NM in 2020.

In addition to the differences in *Simulium* species composition we observed across time, there were significant differences in species composition spatially, with *S. mediovittatum* dominating black fly communities in the northern portion of our study zone and *S. robynae* dominating northern traps. Such spatial partitioning is not uncommon and has been noted in previous studies [38, 39]. Overall, Shannon diversity index (*H*) values were relatively low, ranging from 0.56 to 0.92, compared to other published studies, e.g. [39–43]. This difference is likely attributable to the fact that the Rio Grande runs through desert riparian habitat and is therefore depauperate in species compared to the forested habitat sampled in other studies.

In southern NM, a network of irrigation canals moves water from the Rio Grande to agricultural sites. We detected no significant difference in black fly species composition between Rio Grande sites and farm or irrigation canal sites in 2022. Moreover, our data indicate that river and canal flow, rather than rainfall, dictated black fly abundance. Fredeen et al. found that black fly larval development may be interrupted along irrigation canals in Canada because of transient water flow, suggesting that irrigation canals there are reinfested yearly from larger water sources [44]. Our results suggest a similar process of reinfestation in southern NM.

The source of the reintroduction of black flies each year in southern NM has not been identified. Black flies can overwinter as eggs or larvae and complete development in the spring with the onset of running water [16]. There is evidence of specific lineages of VSNJV over-wintering in the western US, possibly in eggs [21, 45]. Considering the rapid boom in adult abundance following the river release, black fly eggs or larvae in NM must either remain viable throughout the winter or be washed downstream from northern regions of the Rio Grande. To investigate the former possibility, we collected larvae from the surface of dams in southern NM where the slight spillover of water was sufficient to sustain them; these dam faces and adjacent streams were the only sites at which larvae were detected. Intriguingly, these larvae comprised *S. vittatum*, *S. argus* and *S. encisoi*, all three of which are known to occur in NM but had been absent or exceedingly rare in the combined adult collections of this study and that of Young et al. [22]. This discordance suggests that these larvae either fail to produce viable adults or that the adults resulting from these larvae move away from our trapping sites. In support of the former hypothesis, Fonseca and Hart [46] have shown that *S. vittatum* larvae have limited ability to selectively settle in their preferred habitat of fast-moving water and thus may settle at high densities in less favorable habitats. Finally, numerous studies have emphasized the importance of variables affecting the physical and chemical nature of flowing water in black fly larvae presence and development [47–49]. The inconsistent water flow in New Mexico could have an interesting effect on developing black fly communities. However, the source of the adult *S. robynae*, *S. meridionale* and *S. mediovittatum* in southern NM remains to be determined. The strong stratification of the distribution of *S. robynae* suggests an autochthonous origin, likely via overwintering eggs. In contrast, the late expansion of *S. mediovittatum* suggests that this species may move down from the reservoir population in northern NM.

We assessed whether the same environmental factors used by Young et al. [22] were associated with total black fly abundance (summed across all species) in 2021 and 2022; however, no significant associations between total black fly abundance and environmental factors were detected. Shorter river run times and differences in environmental conditions in 2021 and 2022 relative to 2020 may have masked these associations. As previous studies, including our own, have demonstrated associations between various environmental factors and aspects of river morphology with abundance of particular black fly species as well as species diversity, e.g. [39, 42, 50], further interrogation of the impact of such factors on black fly communities and black fly abundance in southern NM is warranted.

## Conclusions

This study supported the hypothesis that certain *Simulium* species, particularly *Simulium robynae*, may be critical to VSV transmission in southern New Mexico, thereby explaining the abrupt cessation of VSV transmission in the summer of 2020. However, we detected no difference in *Simulium* species composition between farm and irrigation canal sites and the Rio Grande, suggesting that the river is the source for black flies along the irrigation network. Intriguingly, the black fly larvae collected from dams were different species than the adults collected in this study. This study further substantiated the impact of human decisions on the timing of the release and restriction of the Rio Grande in southern NM on black fly dynamics and potential for VSV transmission.

### Supplementary Information

**Additional file 1. **NM black fly barcoding metadata. Excel spreadsheet with associated sampling metadata, Geneious Accession Numbers and percentage identity to black fly voucher specimens for all black fly samples used in this project.

**Additional file 2.** Black fly barcode average percent identities to voucher sequences. (a) Minimum, maximum and average sequence identity % ± standard deviation of adult black fly sequences sampled along Rio Grande River in 2020. (b) Minimum, maximum and average sequence identity % ± standard deviation of adult black fly sequences sampled along Rio Grande River, stable and lateral sites in 2022. (c) Minimum, maximum and average sequence identity % ± standard deviation of black fly larvae sequences sampled in Rio Grande River in 2023.

**Additional file 3. **NM complete black fly barcodes. FASTA file containing all black fly barcode sequences (COX1 gene) generated for this study.

**Additional file 4. **Black fly larva phylogeny (2023). Maximum likelihood phylogeny [RaxML v.8, bootstrap values > 75 (1000 replicates) are noted at the nodes] of all larval black fly samples successfully barcoded in NM in 2023, 11 voucher specimen sequences and one outgroup, *Liohippelates pusio*, inferred from mtDNA CoxI.

## Data Availability

The datasets and additional files generated for the current study are available in Genbank & Github: Genbank Accession Numbers: OR458924-OR459501. Github: https://github.com/zhoulhca/Whelpley-Zhou_Black_Fly_Data.git.
