# Supplementary material for: Community composition of black flies during and after the 2020 vesicular stomatitis virus outbreak in Southern New Mexico, USA
Source: Parasit Vectors. 2024 Feb 27;17:93. doi: 10.1186/s13071-024-06127-6 (PMC10900647; doi:10.1186/s13071-024-06127-6)
Supplement: Supplementary file 2 — Additional file 2. Black fly barcode average percent identities to voucher sequences. (a) Minimum, maximum and average sequence identity % ± standard deviation of adult black fly sequences sampled along Rio Grande River in 2020. (b) Minimum, maximum and average sequence identity % ± standard deviation of adult black fly sequences sampled along Rio Grande River, stable and lateral sites in 2022. (c) Minimum, maximum and average sequence identity % ± standard deviation of black fly larvae sequences sampled in Rio Grande River in 2023. [file 13071_2024_6127_MOESM2_ESM.pdf]

a.

	S. meridionale		*S. mediovittatum*		*S. robynae*		*S. griseum/notatum*	
	To Voucher Sequence	Between Samples	To Voucher Sequence	Between Samples	To Voucher Sequence	Between Samples	To Voucher Sequence	Between Samples
**Min**	95.0%	95.1%	95.0%	96.1%	96.7%	95.3%	98.3%	NA
**Max**	99.4%	99.8%	97.2%	99.8%	98.4%	99.7%	98.3%	NA
**Avg**	97.6%	98.0%	96.4%	99.1%	97.8%	98.2%	98.3%	NA
**± SD**	0.7%	0.8%	0.4%	0.5%	0.4%	0.9%	N/A	NA

b.

	S. meridionale		*S. mediovittatum*		*S. robynae*	
	To Voucher Sequence	Between Samples	To Voucher Sequence	Between Samples	To Voucher Sequence	Between Samples
**Min**	94.90%	95.20%	95.10%	96.10%	96.80%	96.80%
**Max**	99.20%	100%	97.60%	99.80%	98.40%	99%
**Avg**	97.70%	97.90%	96.20%	99.00%	97.90%	98.10%
**± SD**	0.70%	0.90%	0.70%	0.80%	0.50%	0.50%

c.

	S. argus		*S. encisoi*		*S. vittatum*	
	**To Voucher Sequence**	Between Samples	To Voucher Sequence	Between Samples	To Voucher Sequence	Between Samples
**Min**	98.10%	97.314	99.842	NA	97.91%	97.30%
**Max**	99.84%	100%	99.842	NA	99.842	100%
**Avg**	98.79%	98.90%	99.842	NA	99.38%	99.13%
**± SD**	0.54%	0.74%	NA	NA	0.48%	0.61

Additional File 5: a) Minimum, maximum, and average sequence identity % ± standard deviation of adult black fly sequences sampled along Rio Grande River in 2020. b) Minimum, maximum, and average sequence identity % ± standard deviation of adult black fly sequences sampled along Rio Grande River, stable and lateral sites in 2022. c) Minimum, maximum, and average sequence identity % ± standard deviation of black fly larvae sequences sampled in Rio Grande River in 2023.
