# Supplementary figures and images for: Community composition of black flies during and after the 2020 vesicular stomatitis virus outbreak in Southern New Mexico, USA
Source: Parasit Vectors. 2024 Feb 27;17:93. doi: 10.1186/s13071-024-06127-6 (PMC10900647; doi:10.1186/s13071-024-06127-6)

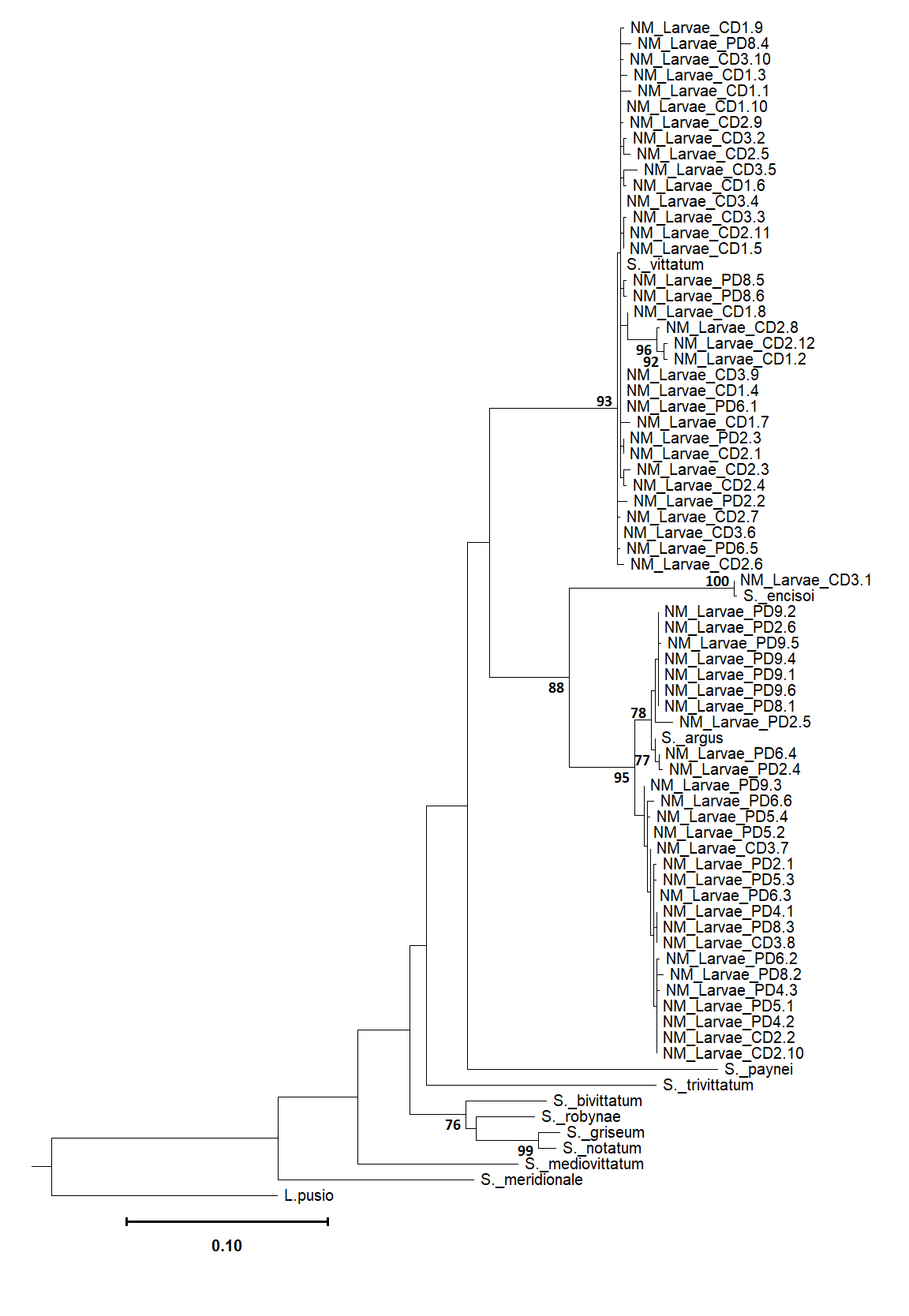

Supplement: Supplementary file 4 — Additional file 4. Black fly larva phylogeny (2023). Maximum likelihood phylogeny [RaxML v.8, bootstrap values > 75 (1000 replicates) are noted at the nodes] of all larval black fly samples successfully barcoded in NM in 2023, 11 voucher specimen sequences and one outgroup, Liohippelates pusio, inferred from mtDNA CoxI. [file 13071_2024_6127_MOESM4_ESM.png]
